# Multimodality imaging of Surgicel^®^, an important mimic of post-operative complication in the thorax

**DOI:** 10.1259/bjro.20210031

**Published:** 2021-09-29

**Authors:** Leonid Roshkovan, Sunil Singhal, Sharyn I Katz, Maya Galperin-Aizenberg

**Affiliations:** ^1^ Department of Radiology, University of Pennsylvania Perelman School of Medicine, Philadelphia, PA, USA; ^2^ Department of Surgery, University of Pennsylvania Perelman School of Medicine, Philadelphia, PA, USA

## Abstract

Absorbable hemostatic agents such as Surgicel are hemostatic materials composed of an oxidized cellulose polymer used to control post-surgical bleeding and cause coagulation. This material is sometimes purposefully left *in situ* where it slowly degrades over time and can produce an imaging appearance that mimics serious post-operative complications such as gangrenous infections and anastomotic leaks as well as potentially mimicking disease recurrence in later stages. In this article, we review the multimodality imaging appearance of this material *in situ* longitudinally in the range of post-operative settings, in order to promote awareness of this entity when interpreting post-operative imaging. We present this as a pictorial review focusing primarily but not exclusively on the chest noting that the thoracic imaging appearance of Surgicel^®^ is less well reported in the published literature. An understanding of this entity may help to minimize erroneous diagnosis of a postoperative complication leading to unnecessary interventions.

## Mechanism of action of Surgicel

**Figure 1.**
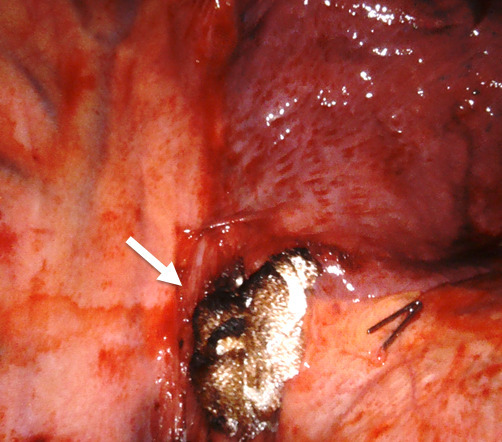
Intraoperative placement of Surgicel. A 74-year-old male undergoing a right lower lobectomy and mediastinal lymph node dissection for lung malignancy. Due to prior radiation therapy to the subcarinal region of the trachea, intraoperative hemorrhage was encountered. The bronchial artery hemorrhage was controlled intraoperatively and to maintain hemostatic control post-operatively, a piece of Surgicel was placed at the site.

Surgicel^®^ is a cellulose-based product that is placed at the site of bleeding intraoperatively to promote hemostasis. This serves as a hemostatic adjunct in the control of local hemorrhage, although the exact mechanism of action is uncertain. When this material saturates with blood, it swells into a brownish or black gelatinous mass, which aids in the formation of a clot (Figure 1).

Several potential mechanisms of action have been proposed for how Surgicel promotes hemostasis.^1,2^ One possible mechanism is that Surgicel acts as a passive agent promoting clot formation by providing a physical scaffold around which platelets can aggregate forming a clot. Alternatively, it is possible that the breakdown of Surgicel produces an acidic environment and that the low pH environment produced by this material promotes clotting.

When left *in situ*, this product slowly degrades over a period of 1–8 weeks^3^ with the pace of degradation influenced by a number of factors including the amount of Surgicel used, the extent of saturation with blood, and the nature of surgical bed. The *in situ* biodegradation occurs through a couple of different mechanisms. The polyuronic acid component of Surgicel is degraded by β-elimination through the activity of glycosidases. In addition, the nonoxidized hydroxyl groups, or fibrous component, are phagocytized and hydrolyzed by macrophages.^4^

## Clinical applications

Most commonly Surgicel is used intraoperatively for rapid control of capillary, venous, and small arterial hemorrhage when ligation or other conventional methods of control are impractical or ineffective. Surgicel comes in sterile-knitted fabric or fibrillar forms and can be sutured or cut to fit the appropriate surgical field.

Once Surgicel is placed into the operative site, effective hemostasis is achieved within 2–8 min following application.^5^ When effective hemostasis is not accomplished after placement of Surgicel into the surgical field, cautery and suction can be performed directly through the material. In some cases, where there is a concern for continued bleeding post-operatively, Surgicel is left in place and allowed to biodegrade *in situ*.

### Multimodality imaging findings associated with retained Surgicel

**Figure 2.**
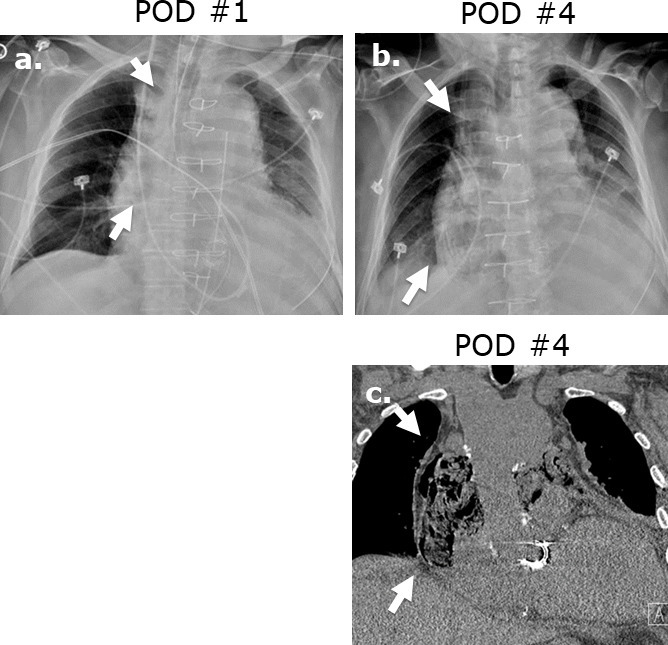
Surgicel mimicking mediastinitis following cardiothoracic surgery. Portable AP chest radiographs were obtained at post-operative day 1 (**a**) and post-operative day 2 (**b**) revealing increasing lucencies in the mediastinum in this 83-year-old male status post-mitral valve and hemiarch aortic repair. Coronal (**c**) image on unenhanced CT performed at post-operative day ^®^ reveals a gas-containing collection in the mediastinum with appearance typical of Surgicel. Correlation with the surgical report confirmed retained Surgicel for post-operative hemostasis.

*Plain radiography:* On radiograph, the presence of Surgicel in the body can produce an appearance that mimics the presence of gas with streaky or mottled lucencies within the operative site. As this material degrades, these lucencies can become initially more prominent mimicking a gas-forming infection or air-leak (Figure 2.).

**Figure 3.**
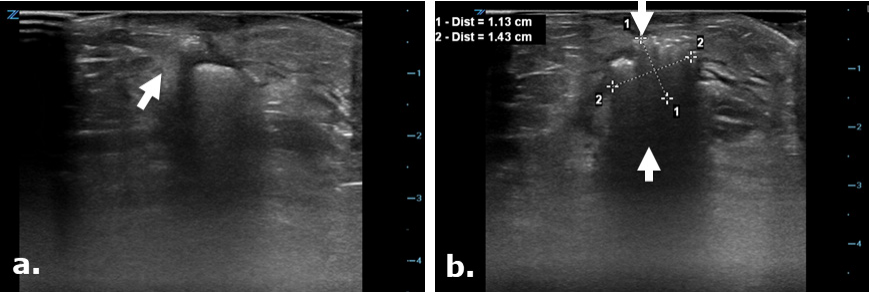
Surgicel in the subcutaneous tissue mimicking a gas containing collection on ultrasound. Focused ultrasound exam images (a, b) were used to evaluate the surgical cut down site following angioplasty in a 75-year-old male. Images depict a focal echogenic mass-like area with posterior acoustic shadowing in keeping with the known Surgicel that was retained post-operatively for hemostasis. This appearance closely mimics an air containing collection.

*Ultrasonography:* On ultrasound, the presence of Surgicel in a wound typically appears as a complex mass-like structure with heterogeneous areas of internal hypoechoic and hyperechoic textures (Figure 3). The hypoechoic material is attributed to gelatinous mass formed from the saturation of the Surgicel fabric with blood. The echogenic component of this appearance is attributed to trapped air within the gelatinous matrix. In addition, serosanguinous fluid may be present surrounding the Surgicel mass-like structure. The sonographic appearance of Surgicel can mimic an abscess due to gas-forming organisms or retained surgical sponges.^6^

**Figure 4.**
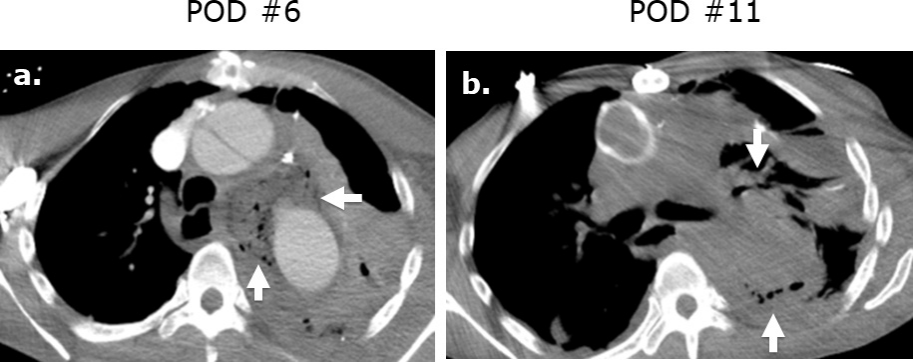
Surgicel mimicking mediastinitis following cardiothoracic surgery on CT. Axial (**a**) intravenous contrast-enhanced CT following repair of a type A aortic dissection in a patient with leukocytosis at post-operative day ^®^ and demonstrates a large gas-containing collection. Correlation with the surgical note confirmed that Surgicel was retained post-operatively for hemostasis. A follow-up axial unenhanced CT (**b**) obtained at post-operative day 11 demonstrates interval partial resorption of the Surgicel.

**Figure 5.**
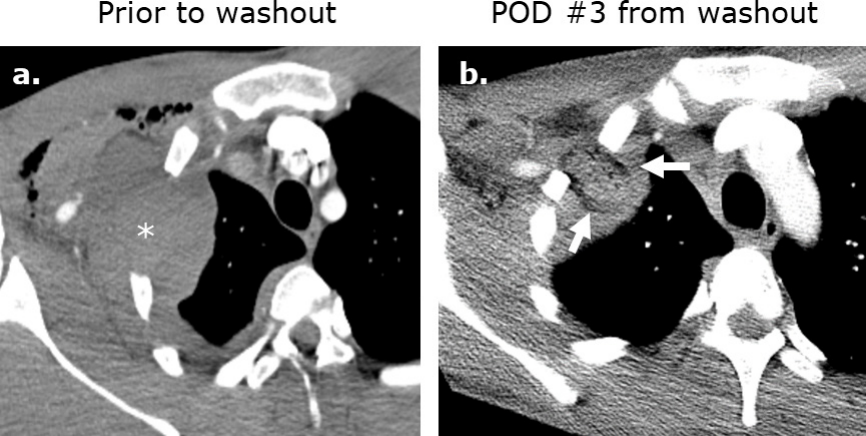
Surgicel mimicking abscess formation following washout of an iatrogenic hematoma. (**a**) Axial intravenous-enhanced CT image obtained at post-operative day 9 following ^®st^ rib resection for thoracic outlet syndrome in a 19-year-old male with pain and tachycardia revealing a hematoma at the surgical site. Axial (**b**) and coronal (**c**) intravenous contrast-enhanced images obtained following washout of the hematoma reveals Surgicel in the operative bed.

**Figure 6.**
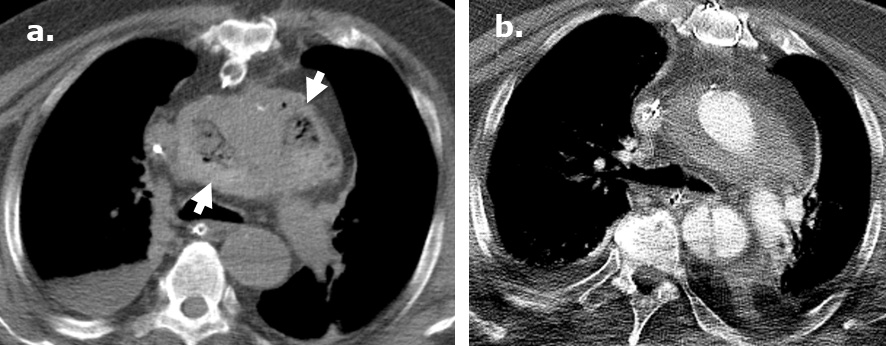
Longitudinal postoperative changes of Surgicel following type A aortic dissection repair. An axial image from an unenhanced CT (**a**) performed in a 71-year-old male at post-operative day ^®^ following a type A dissection repair demonstrates a heterogeneous gas-containing collection surrounding the aortic root in keeping with Surgicel. Subsequent axial intravenous-enhanced CT (**b**) performed at post-operative day 11 reveals near resolution of the Surgicel with evolution to a homogenous perigraft collection typically seen in the post-operative setting.

**Figure 7.**
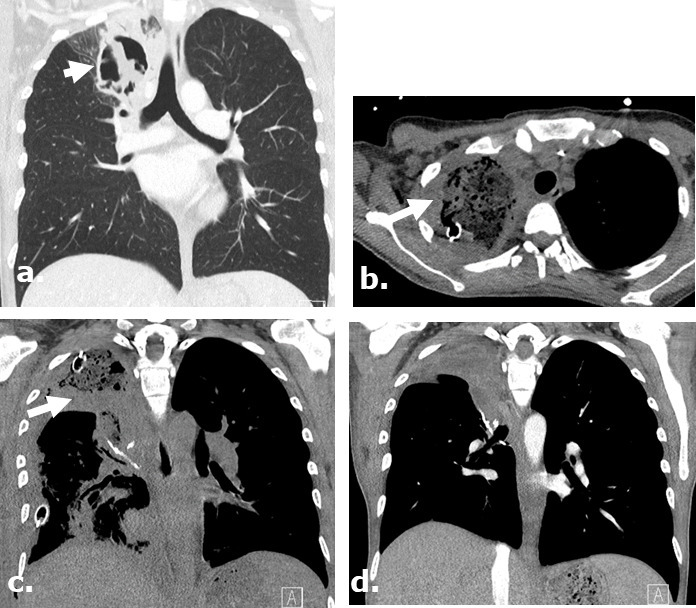
Surgicel in the post-operative bed mimicking infection following a lobectomy on CT. A 37-year-old female with AML and aspergilloma. Surgicel was used intraoperatively for hemostasis. Coronal (**a**) unenhanced CT images demonstrate an aspergilloma in the right upper lobe of the lung. Axial and coronal (b., (**c**) unenhanced CT images obtained 4 days post-surgery demonstrate large surgical cavity filled with low attenuation material mimicking a gas-containing infectious collection which was confirmed to be Surgicel on the operational note. Coronal (**d**) unenhanced CT images obtained 2 months after surgery demonstrate resolution of the finding.

**Figure 8.**
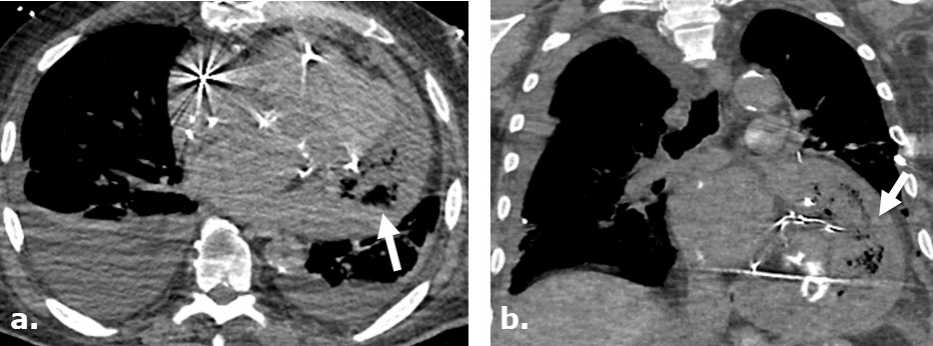
Surgicel mimicking infected pericardial fluid following cardiothoracic surgery complicated by sepsis and shock. Axial (**a**) and coronal (**b**) non-contrast-enhanced CT images of a 75-year-old male following pericardial washout and irrigation of an infected pericardial effusion demonstrates large complex gas-containing pericardial effusion, mimicking infected collection. Surgical note indicates intraoperative utilization of Surgicel for hemostasis.

*Computed tomography:* Surgicel can have several patterns of appearance on CT. The most common is an appearance of focal collections of gas within mixed-attenuation masses (Figures 4–8). This material can also have the appearance of small punctate areas of gas adjacent to a larger globular gas collection, or less commonly, a linear pattern of gas within a soft tissue attenuation mass-like structure. Typically, there are no internal air-fluid levels, central or peripheral enhancement associated with Surgicel on CT.^7^

Differentiating between Surgicel and post-operative complications can be challenging and most commonly encountered on CT mimicking postoperative hematoma, abscess^8^ or inadvertently retained surgical foreign body. These other post-operative conditions are often suggested as potential differential diagnostic possibilities in routine clinical practice by radiologists not aware of the use of Surgicel during the surgical procedure or not familiar with appearance of Surgicel on CT. Frati et al^9^ demonstrated that in the post-operative setting, the ability to differentiate Surgicel from a collection or an abscess on CT, while withholding clinical history about the use of Surgicel, resulted in correct interpretation in only 11% of the cases.

Several clinical clues and imaging features can assist in the correct identification of Surgicel on post-operative chest CT, and differentiation from other potential post-operative complications. For example, the imaging findings associated with post-operative infection include complex fluid collections, often with peripheral rim enhancement following injection of intravenous contrast. When the infection is on the basis of a gas-forming organism, there can be gas interspersed within these complex collections that is visible on CT. The appearance of gas and fluid collections in the operative site is less likely to be due to infection very early in the post-operative period (0–3 days), but becomes more concerning for infection with increasing time in the perioperative setting.^10^ In distinguishing between post-operative infection and retained Surgicel, the latter typically does not demonstrate rim enhancement on IV contrast-enhanced CT. However, the absence of rim enhancement around a post-operative collection alone is not reliable to exclude the possibility of infection and if the use of Surgicel is suspected in imaging, correlation with the surgical history is recommended.

**Figure 9.**
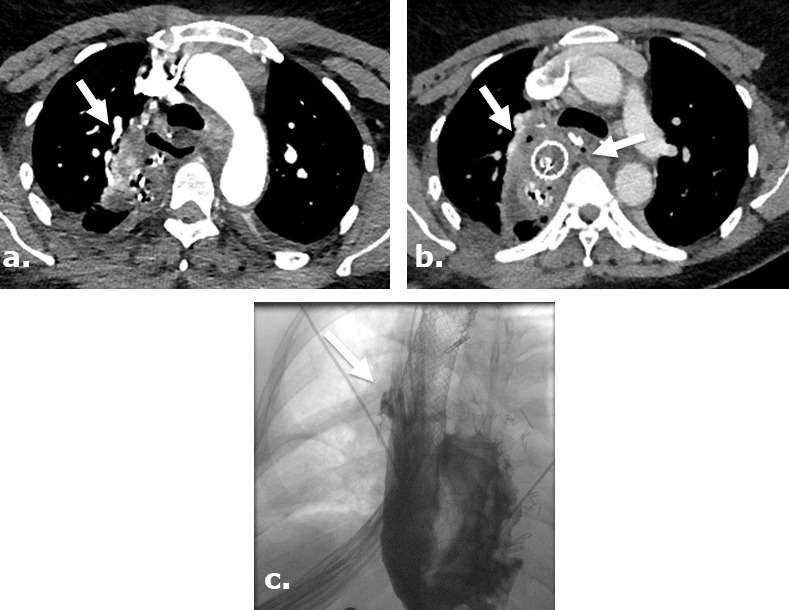
Anastomotic leak after Ivor Lewis esophagogastrectomy in a surgical case where Surgicel was not used. Axial images of a contrast-enhanced CT obtained 5 days after surgery (**a**) in a 46-year-old female with a history of stage I esophageal adenocarcinoma, demonstrates extra luminal gas anterior to the enteric tube. An axial view of contrast-enhanced CT obtained 9 days after surgery (**b**) demonstrates persistent gas and fluid surrounding the now stented gastric pull through. Single upper gastrointestinal series with water-soluble contrast obtained 13 days after surgery (**c**) demonstrates persistent leak at the level of esophagogastric anastomosis.

Another thoracic post-operative complication that may appear similar to Surgicel on imaging are anastomotic leaks, which may lead to serious morbidity and mortality. For example, anastomotic leaks following esophagectomy, reported in up to 25% of esophagectomies, carry an associated mortality as high as 20%,^11^ usually within the first 10 days after surgery. On CT imaging, gas that escapes the gastrointestinal lumen into the mediastinum will typically collect near the surgical site, often in association with fluid, an imaging appearance that overlaps with that of Surgicel (Figure 9). Imaging characteristics that can help to differentiate between an anastomotic leak and Surgicel on CT include evidence of esophageal wall discontinuity or a fistulous tract extending from the surgical site. Fluoroscopy with oral contrast can be helpful in confirming the presence of an anastomotic leak.^12^

**Figure 10.**
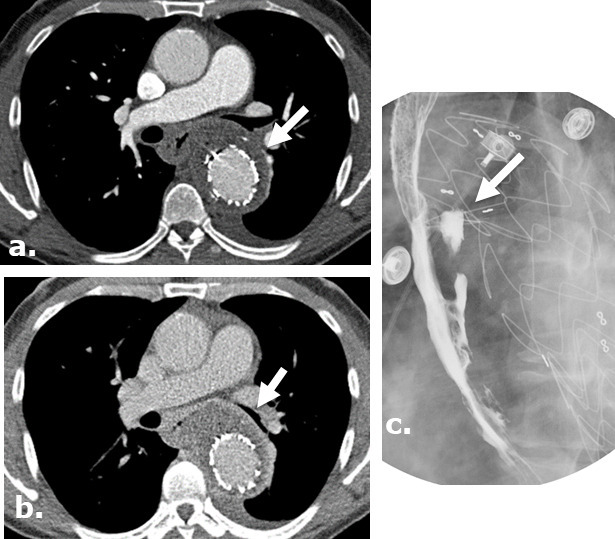
Aorto-esophageal fistula, leading to mediastinal abscess in a surgical case where Surgicel was not used. Arterial (**a**) and delayed (**b**) phases of CTA of the chest of a 56-year-old male demonstrate perigraft gas-containing fluid collection containing small gas bubbles. There is esophageal wall thickening and loss of the fat plane between the aorta and esophagus. A barium swallow study demonstrates extravasation of barium toward the aortic stent, consistent with aorto-esophageal fistula (**c**).). Patient was treated with antibiotics and G-tube placement.

Finally, serosanguinous fluid collections are commonly observed on CT in the post-operative setting, can persist for years and may be confused with Surgicel especially in the early perioperative period. For example, following open aortic repair perigraft fluid is extremely common in the early post-operative period and can persist for up to at least one year. This serosanguinous fluid is typically high in attenuation on CT (50–80 HU) immediately post-op, due to the presence of acute blood products, and becomes progressively lower in attenuation on CT to approach the range of complex fluid (20–40 HU) as these blood products evolve. This fluid is typically relatively homogeneous in appearance on CT and is often seen in association with a small amount of gas post-operatively, with lingering small locules of gas sometimes present up to 3 months following surgery (Figure 10).^13^ Surgicel may mimic this appearance although typically has more associated gas than is seen within serosanguinous perigraft collections in the perioperative period.

**Figure 11.**
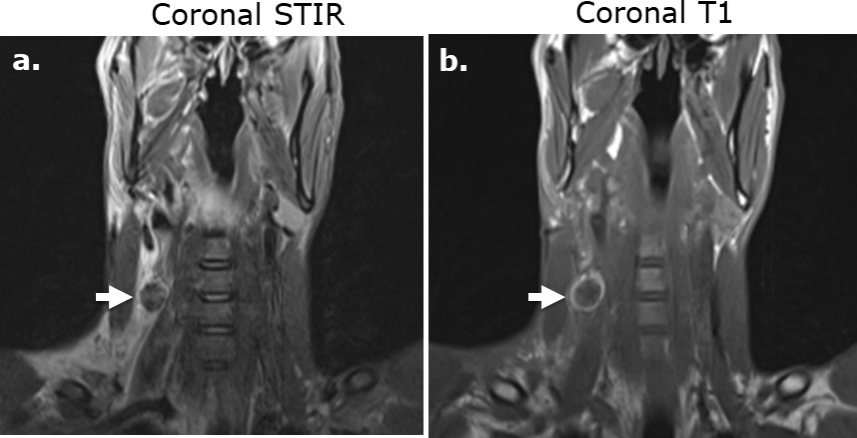
Subcutaneous Surgicel collection following stab wound injury and superficial dissection on MRI. Coronal STIR (**a**) and coronal T1-weighed (**b**).) images of the neck in a 29-year-old male following a stab wound injury with Surgicel retained at the surgical site to promote hemostasis. The Surgicel material is nearly uniformly low in *T*_2_-weighted signal intensity relative to fluid (**a**) and demonstrates a rim of high signal intensity relative to muscle on unenhanced *T*_1_-weighted imaging (**b**).

**Figure 12.**
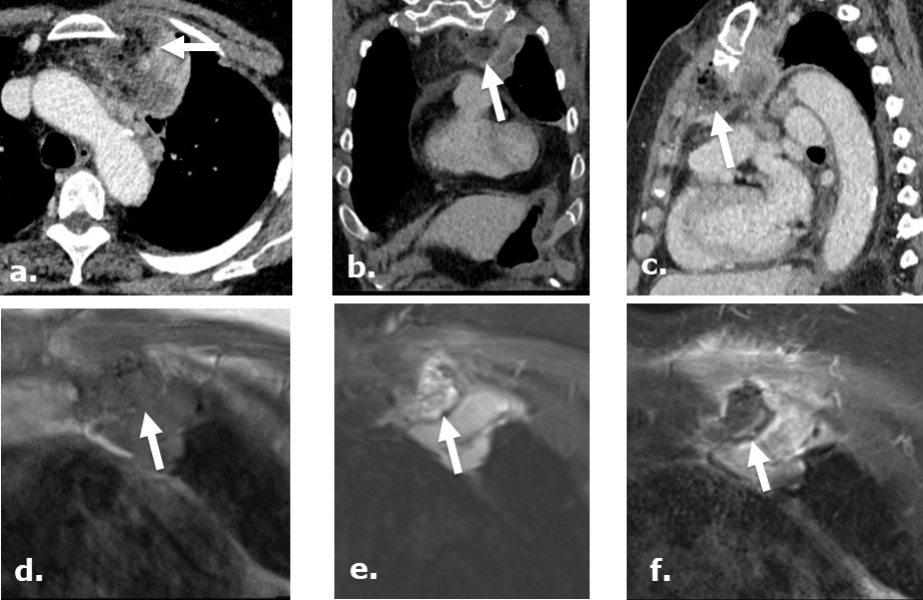
A 75-year-old female post-biopsy of a mediastinal mass. Contrast-enhanced CT images axial (**a**), coronal (**b**) and sagittal (**c**), day 7 post-biopsy, demonstrate heterogeneous, complex gas-containing collection medial to a biopsied mass, referring physician has confirmed the use of Surgicel during the procedure in this location. MRI images of the same region obtained status post biopsy day 12 demonstrate a collection isointense to muscle on coronal unenhanced T1 (**d**) and heterogeneous intensity focus on coronal STIR (**e**) with no enhancement in the retained Surgicel on coronal gadolinium enhanced T1-weighed (**f**). There are punctuate low intensity foci in the collection compatible with gas while surrounding chest wall and mass demonstrate enhancement.

*Magnetic resonance imaging:* On MRI, Surgicel material is typically heterogeneous and hypointense to fluid on *T*_2_-weighted imaging, which differentiates it from abscess formation which is typically hyperintense to fluid on *T*_2_-weighted imaging (Figure 11). In addition, regions of *in situ* post-operative Surgicel can be encircled by a rim that is hyperintense to muscle on non-intravenously enhanced *T*_1_-weighted imaging (Figures 11 and 12). There are reports in the literature of streak-like enhancement on intravenously enhanced imaging associated with Surgicel in the operative bed in following neurooncologic surgery which is hypothesized to reflect granuloma formation but can be mistaken for local recurrence.^14^

## Post-operative complications associated with Surgicel

While Surgicel is frequently used safely in the operative setting, there have been some reports of complications secondary to use of this material. For example, the swelling and mass effect exerted by Surgicel has been reported to cause nerve compression potentially leading to paraplegia after thoracotomies.^15^ In addition, Surgicel can induce the formation of granulomas, which can result in unnecessary surgical intervention. For example in the brain, granuloma formation associated with Surgical^®^ has been reported to mimic tumor recurrence resulting in a subsequent surgical intervention to exclude tumor recurrence.^16^ As a result, Surgicel is typically removed from the surgical field whenever possible after hemostasis has been achieved.

## Conclusions

In summary, Surgicel is commonly used in the surgical setting and is sometimes intentionally left *in situ* to promote hemostasis in the post-operative setting. Due to similarities in imaging features of post-operative complications, Surgicel can be mistaken for potentially serious entities including post-surgical infection, anastomotic leaks and, in the brain, local recurrence of malignancy. Correlation with surgical operative notes, clinical data and knowledge of the expected imaging appearance of this material, and those of the entities with which it can be confused, can potentially prevent unnecessary intervention.
